# Peste des Petits Ruminants Risk Factors and Space-Time Clusters in Bangladesh

**DOI:** 10.3389/fvets.2020.572432

**Published:** 2021-01-25

**Authors:** A. K. M. Anisur Rahman, Sk Shaheenur Islam, Md. Abu Sufian, Md. Hasanuzzaman Talukder, Michael P. Ward, Beatriz Martínez-López

**Affiliations:** ^1^Department of Medicine, Bangladesh Agricultural University, Mymensingh, Bangladesh; ^2^Department of Livestock Services, Krishi Khamar Sarak, Dhaka, Bangladesh; ^3^Department of Parasitology, Bangladesh Agricultural University, Mymensingh, Bangladesh; ^4^Sydney School of Veterinary Science, The University of Sydney, Camden, NSW, Australia; ^5^Department of Medicine and Epidemiology, Center for Animal Disease Modeling and Surveillance, School of Veterinary Medicine, University of California, Davis, Davis, CA, United States

**Keywords:** PPR, cluster analysis, risk mapping, risk-based surveillance, goats, sheep

## Abstract

Peste des Petits Ruminants (PPR) is endemic in Bangladesh, but its spatial distribution and risk factors have not yet been reported. Using four years of national-level, passive surveillance data (2014 to 2017), in this study we aimed to identify risk factors, create PPR risk maps and describe PPR time-space clusters. We selected PPR case records—mainly based on presumptive diagnosis of small ruminants in subdistrict veterinary hospitals—and sheep and goat population data from all 64 districts of Bangladesh. Peste des Petits Ruminants cumulative incidence per 10,000 animals at risk per district was used to conduct cluster and hotspot analysis and create predictive maps for each year and all 4 years combined. The association between PPR cumulative incidence and hypothesized risk factors—including climatic variables, elevation, road length, river length, railroad length, land cover, and water bodies—was analyzed using a geographically weighted regression model. The total number of PPR cases reported during the study period was 5.2 million. We found that most PPR cases (27.6%) were reported in the monsoon season. The highest and lowest proportions of cases were reported from Rajshahi (36.1%) and Barisal divisions (2.1%), respectively. We identified five space-time clusters, 9 high–high clusters, and 9 hotspots. The predicted cumulative incidences of PPR were persistently higher in north-east, north-west, and south-east parts of Bangladesh. Road length (*P* = 0.03) was positively associated with PPR incidence in Bangladesh. Results suggest that movement of animals (road length) plays an important role in the epidemiology of PPR in Bangladesh. Along with restriction of animal movement, hotspots and high–high clusters should be targeted first for immunization coverage in Bangladesh and similar PPR endemic countries to achieve eradication.

## Introduction

Peste des Petits Ruminants (PPR) is an extremely contagious, devastating, and economically important transboundary viral disease of small ruminants (1). It is highly endemic in Africa, the middle East, and Asia including Bangladesh (2, 3). Globally, nearly two-thirds of all small ruminants are at risk of PPR (4). Fever, ocular and nasal discharge, stomatitis, and profuse diarrhea are the characteristic clinical signs of PPR (5). PPR-infected animals shed large quantities of virus (PPRV) through nasal and ocular discharges, saliva, and feces. The PPRV is fragile outside the host and is rapidly destroyed by heat and sunlight (6). Transmission of the disease can occur through ingestion of contaminated feed and water in addition to the respiratory mucosal route in animals within close proximity. The morbidity of PPR can be as high as 100%, and the fatality rate can exceed 90% in unexposed population (7). The annual global economic loss attributed to PPR was estimated to be US$1.2–1.7 billion, as a result of mortality, production loss, and control costs (8). With a benefit-cost ratio of 33.8, the net benefit of global eradication of PPR is estimated to be US$74.2 billion (2). Control of PPR is relatively easier than for foot-and-mouth disease (seven serotypes and vaccine using one serotype does not protect against infection with the other serotypes) due to viral antigenic stability, presence of a single virus serotype, and the induction of a lifelong immune response after vaccination (6). Considering this, the World Organization for Animal Health (OIE) and the Food and Agriculture Organization (FAO) have targeted for PPR eradication globally by 2030 (8).

Bangladesh has 29.5 million (26 million goats and 3.5 million sheep) small ruminants (9). Among goat breeds in Bangladesh, 90% of the population is Black Bengal; the majority of sheep breed is Native Bengal (10). More than 98% of small ruminants are managed by small, marginal, and landless poor farmers (11). Small ruminants play an important role in household economies as a ready source of cash and protein. Being one of the main constraints to small ruminant production and welfare, PPR (goat plague) threatens the food security and livelihood of the poorest communities in Bangladesh. Actual annual losses due to PPR in Bangladesh is not known but predicted to be US$25 million (12). In Bangladesh, PPR morbidity, mortality and case fatality has been estimated to be 75%, 59%, and 78%, respectively (13). Peste des Petits Ruminants outbreaks occur regularly in different districts of Bangladesh, and the risk was reported to be significantly higher in monsoon and winter seasons, in the Jamunapari breed and in older goats (13–15). There is a huge demand in Bangladesh for goat meat due to its taste and palatability. In addition, Bangladesh is now exporting small quantities of goat meat to a few countries; however, the presence of PPR hinders export at a larger scale.

Peste des Petits Ruminants vaccines are available (~5 million doses) in Bangladesh. Bangladesh has drafted a National Strategic Plan (NSP) within the context of the global strategy for the control and eradication of PPR. The NSP is awaiting approval. There has been some progress as a part of the control program, including targeted mass vaccination, increased farmers' awareness, and engagement in the program in some goat rearing districts (Jashore, Chuadanga, Meherpur, and Kustia); however, the vaccination coverage is unachieved in other goat-rearing districts. Sub-district and district veterinary hospitals supply a limited amount of PPR vaccine, mostly only purchased by conscientious farmers; however, there are sporadic vaccination programs to create awareness among farmers. Knowledge on disease hotspots and risk factors will enable policy-makers to plan efficient control measures in Bangladesh (16), however such knowledge is lacking. In this study we aimed to identify PPR time-space clusters, determine risk factors and develop predictive PPR risk maps. We used four years of national level, passive surveillance data to achieve these aims.

## Materials and Methods

### Data

#### PPR Case Data

There are 8 divisions, 64 districts and 491 sub-districts (or upazilas) in Bangladesh. Livestock farmers report disease by visiting—with or without their animals—upazila veterinary hospitals (UVH). As a part of routine disease surveillance, the Department of Livestock Service (DLS) collects data on every case attending an UVH. Subdistricts routinely collect and report surveillance data monthly to districts and divisions. Divisions then collate and forward data to the Epidemiology Unit of DLS. Peste des Petits Ruminants cases in goats and sheep between 2014 and 2017 were obtained from the DLS and used for this study. The diagnosis of these cases was based mainly on the characteristic clinical signs of fever, ocular and nasal discharge, stomatitis, and profuse diarrhea (5). To increase the positive predictive value of a presumptive PPR diagnosis, UVH used all of these five clinical signs which characterize PPR. Other diseases which produce all of these five clinical signs are not present in small ruminants in Bangladesh. The laboratory system in Bangladesh comprises 500 veterinary hospitals at the subdistrict and municipality levels, one Central Disease Investigation Laboratory (CDIL), 9 Field Disease Investigation Laboratories (FDILs), and one National Reference Laboratory for PPR [SAARC Regional Leading Diagnostic Laboratory at Bangladesh Livestock Research Institute (BLRI) for PPR (PPR-RLDL) and the National Institute of Biotechnology (NIB)]. Clinical samples or samples from outbreak investigations are initially tested at FDILs. Field Disease Investigation Laboratories send samples to CDIL for further confirmation using molecular techniques. Finally, CDIL sends samples to the PPR-RLDL and NIB for advanced diagnosis (molecular diagnosis including sequencing and virus isolation). Approximately 1% of passive surveillance cases were confirmed through basic (cELISA) and molecular tests (RT-PCR and sequencing). The data used in this study contained information on case date and district name. The small ruminant population data in each district were collected from DLS.

#### Environmental and Climatic Data

We downloaded (~1 km^2^ resolution) monthly average temperature (°C), precipitation (mm), solar radiation (kJ m^−2^ day^−1^), and wind speed (m s^−1^) data from world climate data (www.worldclim.org). Data on road length, railroad and river, country mask elevation, and land cover were also downloaded (www.diva-gis.org). The method of extracting Bangladesh district-specific climate and geographical data has previously been described (17). Seasonal data were created by aggregating monthly data: winter (December–February), pre-monsoon (March–May), monsoon (June–August), and post-monsoon (September–November) (17). For the geographically weighted regression analysis, a Bangladesh district shapefile with log-transformed cumulative incidence of PPR and climate and geographical data was used.

### Analysis

#### Descriptive Statistics

The PPR database was transferred to R 3.6.1 (18) for descriptive analysis. The “aggregate” and “summary” functions in the “stats” and “base” packages, respectively, were used to aggregate and summarize the data by month, season, and year.

#### Spatial Analysis

##### Cluster Analyses

The outcome of interest in spatial analysis was district cumulative incidence of PPR per 10,000 small ruminants. A global cluster method (Moran's I) was used as a first step to determine if clustering was present in the study region (19). Then, three different methods (local indicators of spatial association, Getis Ord, and SaTScan) were applied to determine the locations of the clusters. We used three different clustering methods as local indicators of spatial association (20) detects outliers efficiently (21), Getis Ord (22) identifies locations surrounded by a cluster of high or low values, and SaTScan (23) detects small, compact clusters both in space and time (24). The details about these methods have been described previously (17).

We used the empirical Bayesian kriging (EBK) method for geostatistical prediction and generation of risk maps (Geostatistical Analyst, Geostatistical Wizard, ArcGIS 10.7.1, Environmental System Research Institute, USA). We created risk maps for each year to interpolate PPR incidence throughout Bangladesh (25).

##### Geographically Weighted Regression

The geographically weighted regression (GWR) was performed in GeoDa 1.14.0 (26) using log-transformed PPR cumulative incidence as the dependent variable in the model and climate and geographical variables as predictors. Univariable GWR models were used to screen risk factors (*P* < 0.10) and a final multivariable GWR model was used to the best set of risk factors (*P* < 0.05) that predicted log-transformed PPR cumulative incidence. The detailed analytical approach has been described previously (17).

## Results

### Descriptive Statistics

Overall, the median annual [interquartile range (IQR)] average small ruminant population per district was 244,547 (171,019–415,391). Between 2014 and 2017, there was a total of 524,805 PPR cases reported from all 64 districts (Table 1), and the median (IQR) PPR cumulative incidence (per 10,000 small ruminants) per district in Bangladesh was 198 (98–313). There were 116,514 PPR cases in 2014; 88,258 in 2015; 130,517 in 2016; and 189,516 in 2017. The median (IQR) number of PPR cases per district were 82 (35–202) in 2014; 57 (17–136) in 2015; 68 (30–173) in 2016; and 98 (26–274) in 2017. The highest proportion of PPR cases were reported in June (9.5%), during the monsoon season (27.6%), and from Rajshahi division (36.2%) (Table 1).

**Table 1:** Distribution of Peste des Petits Ruminants cases based on passive surveillance data reported from 64 districts in Bangladesh during 2014–2017.

**Variable**	**Cases (average cumulative incidence per 10,000 small ruminants)**	**% (95% confidence interval)**
**Year**		
2014	116,514 (50.8)	22.2 (22.1–22.3)
2015	88,258 (45.9)	16.8 (16.7–16.9)
2016	130,517 (65.8)	24.9 (24.8–25.0)
2017	189,516 (84.1)	36.1 (35.9–36.2)
**Month**		
December	43,577	8.3 (8.2–8.4)
January	35,308	6.7 (6.6–6.8)
February	35,633	6.8 (6.7–6.9)
Winter (December–February)	114,518	21.8 (21.7–21.9)
March	38,833	7.4 (7.3–7.5)
April	43,958	8.4 (8.3–8.5)
May	48,255	9.2 (9.1–9.3)
Pre-monsoon (March–May)	131,046	24.9 (24.8–25.1)
June	49,980	**9.5** (9.4–9.6)
July	45,257	8.6 (8.5–8.7)
August	49,469	9.4 (9.3–9.5)
Monsoon (June–August)	144,706	**27.6** (27.5–27.7)
September	48,879	9.3 (9.2–9.4)
October	44,906	8.5 (8.4–8.6)
November	40,750	7.7 (7.6–7.8)
Post-monsoon (September–November)	134,535	25.6 (25.5–25.7)
**Division**		
Barisal	10,839 (6.9)	2.1 (2.0–2.2)
Chittagong	90,512 (53.4)	17.2 (17.1–17.3)
Dhaka	48,586 (29.5)	9.2 (9.1–9.3)
Khulna	37,781 (20.8)	7.2 (7.1–7.3)
Mymensingh	14,645 (11.7)	2.8 (2.7–2.9)
Rajshahi	190,052 (100.5)	36.2 (36.1–36.3)
Rangpur	92,412 (55.0)	17.6 (17.5–17.7)
Sylhet	39,978 (29.7)	7.6 (7.5–7.7)
Total	524,805	100

*Bold values indicated highest and lowest percentage of cases*.

### Clustering and Risk Prediction

**Figure 1:**
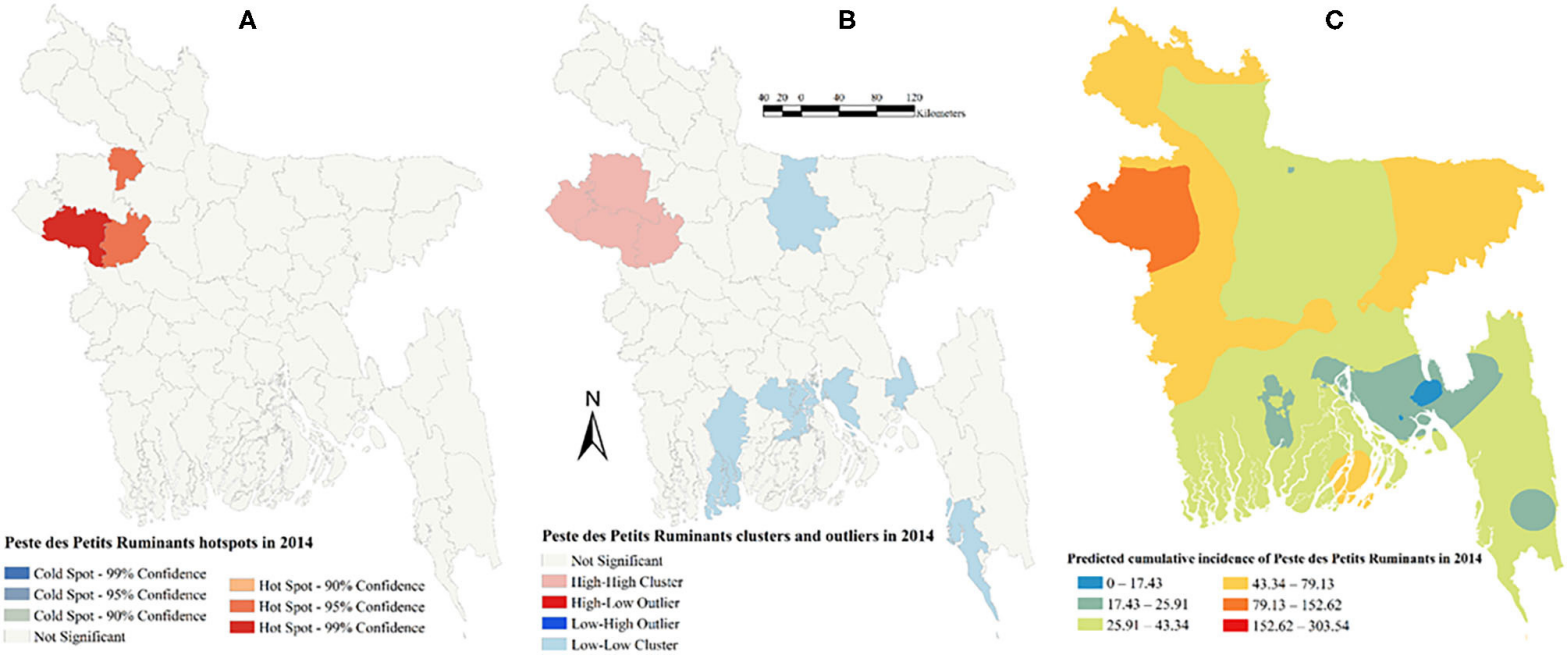
Peste des Petits Ruminants hotspots **(A)**, clusters and outliers **(B)**, and risk map **(C)** in 2014.

**Figure 2:**
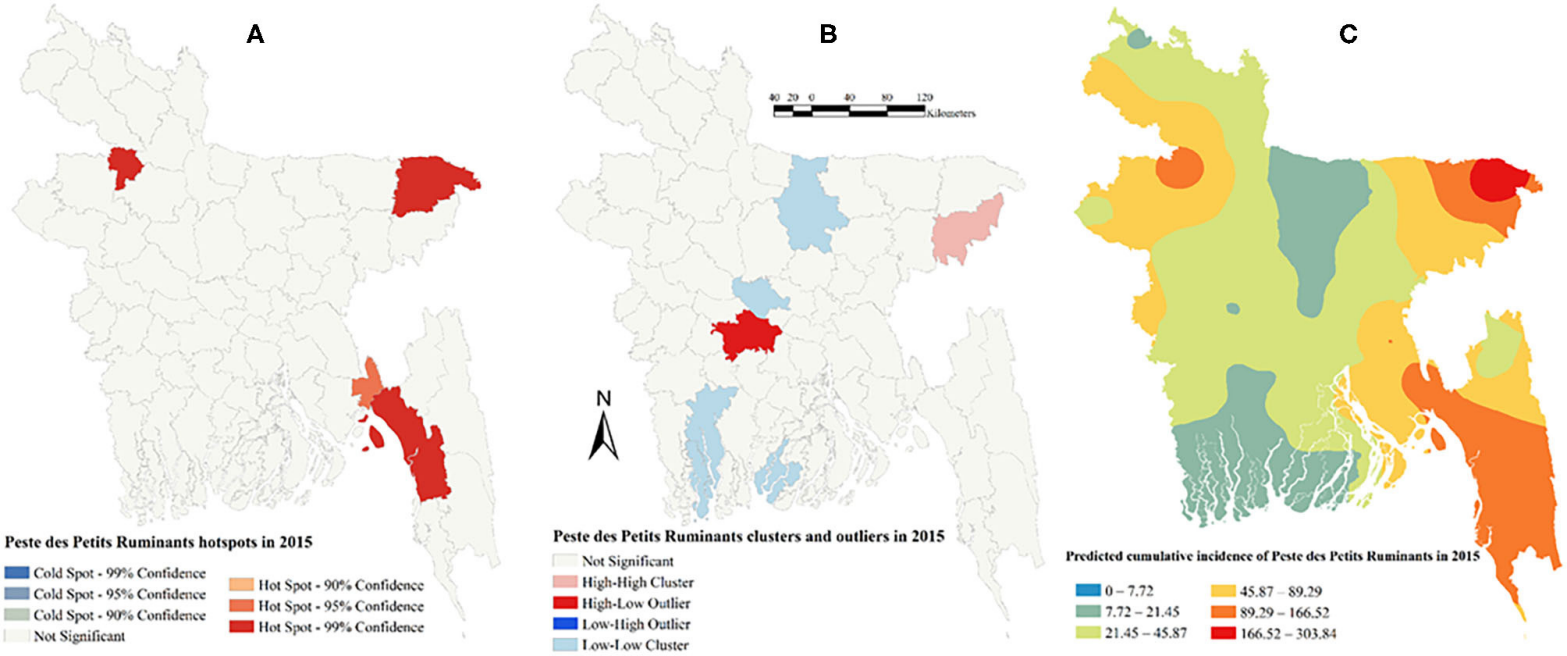
Peste des Petits Ruminants hotspots **(A)**, clusters and outliers **(B)**, and risk map **(C)** in 2015.

**Figure 3:**
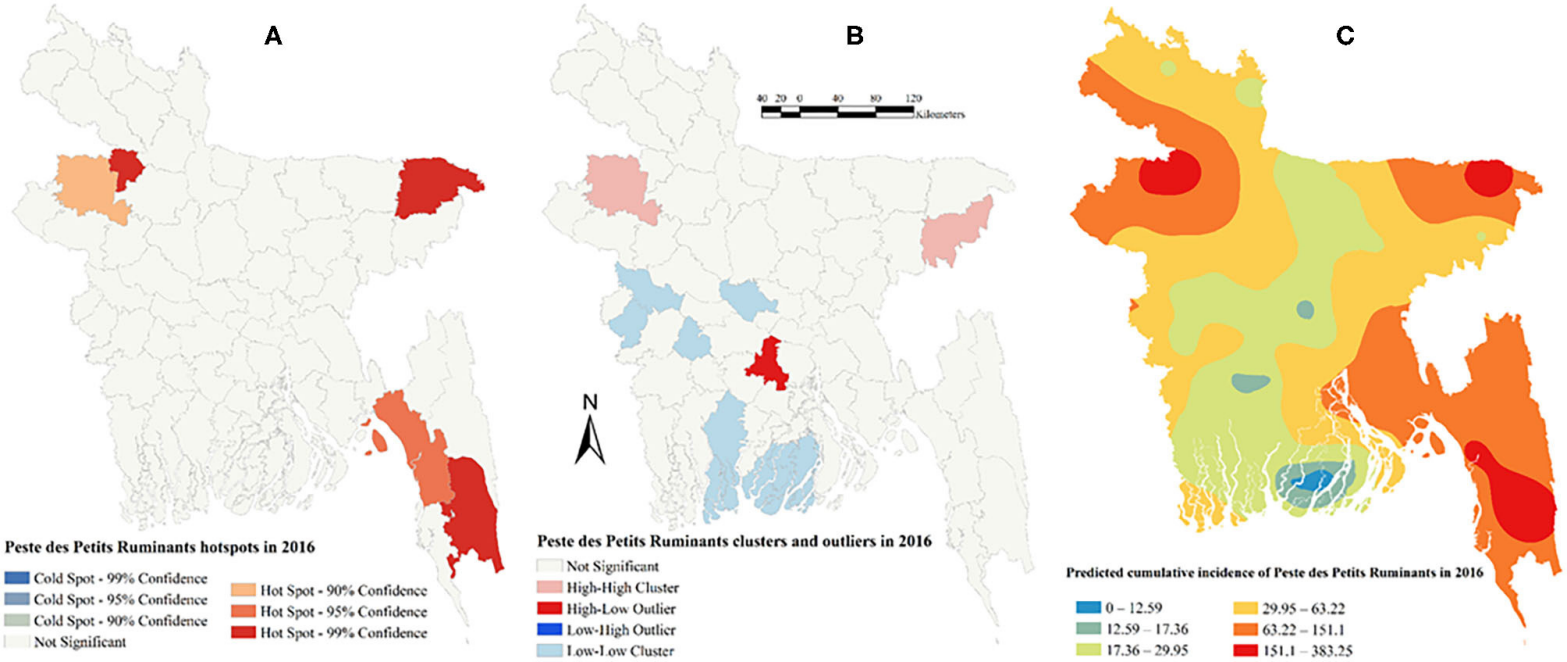
Peste des Petits Ruminants hotspots **(A)**, clusters and outliers **(B)**, and risk map **(C)** in 2016.

**Figure 4:**
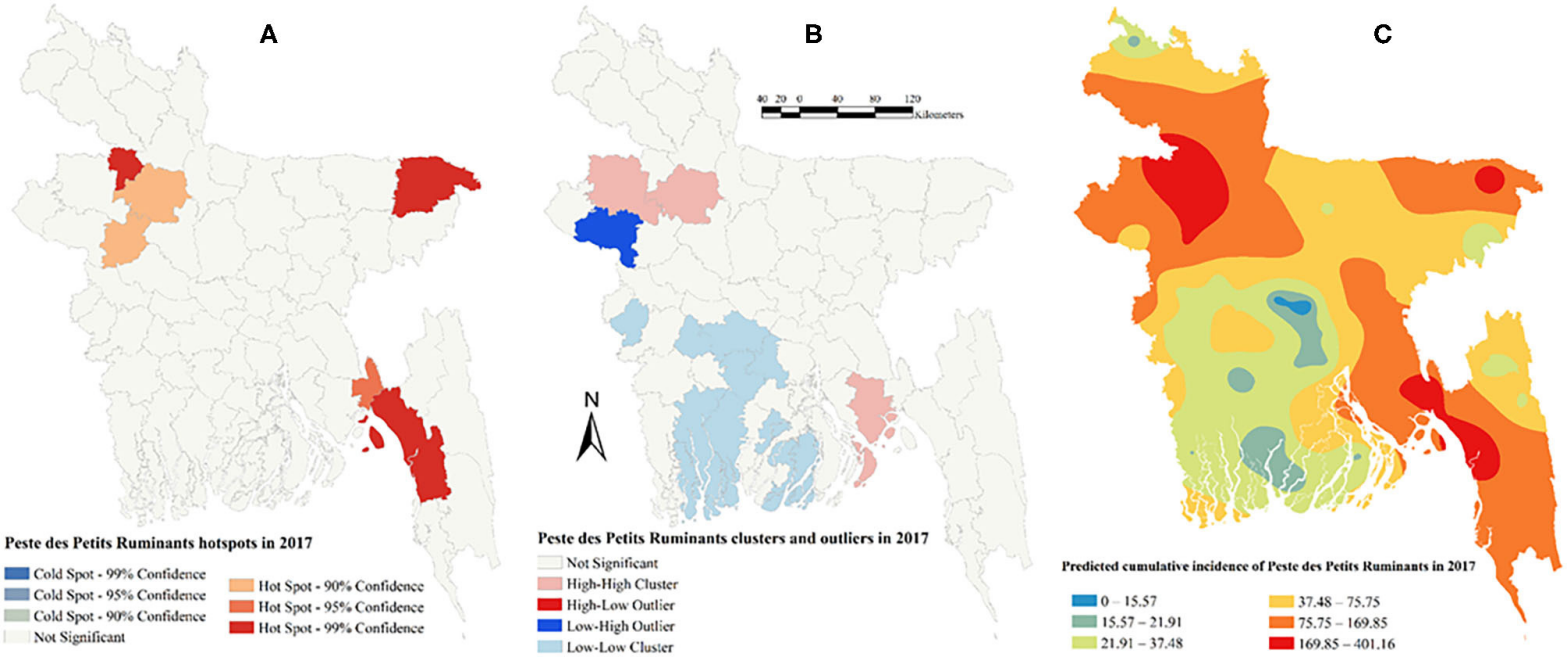
Peste des Petits Ruminants hotspots **(A)**, clusters and outliers **(B)**, and risk map **(C)** in 2017.

**Figure 5:**
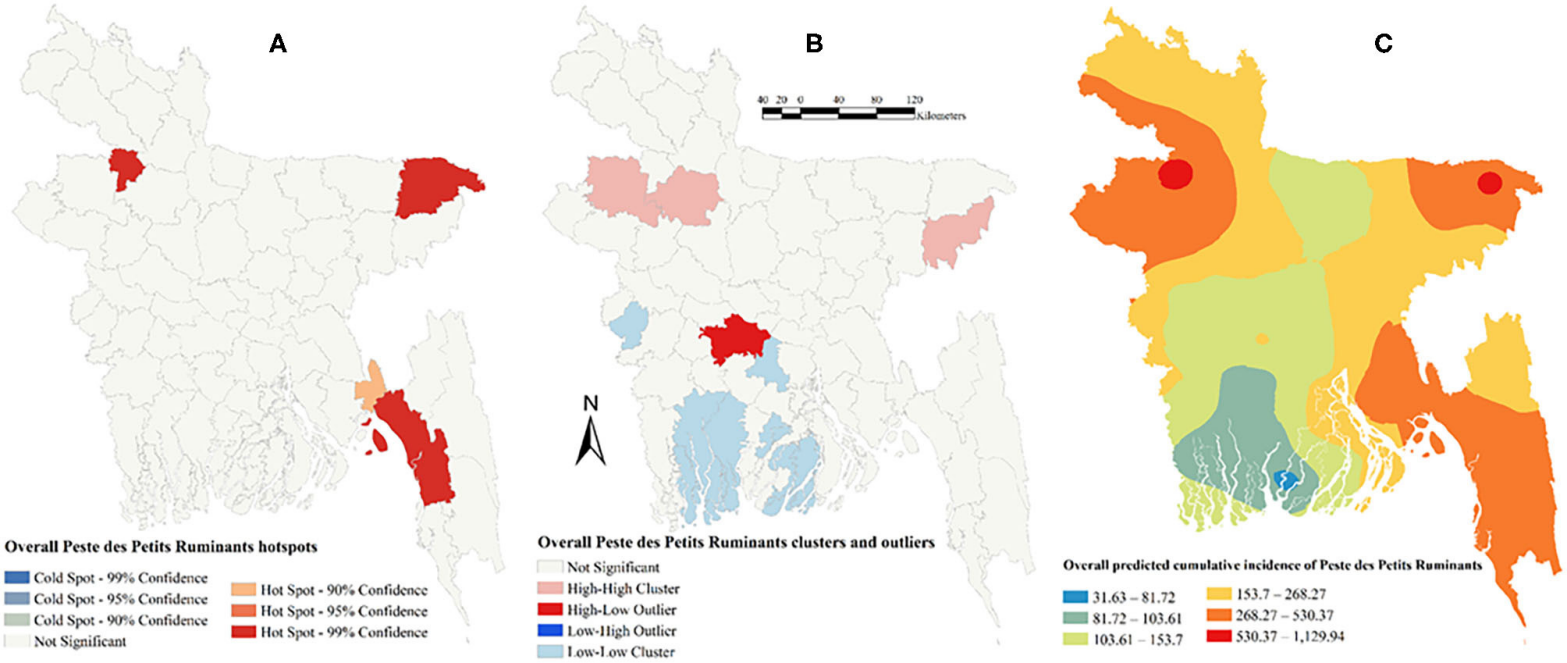
Peste des Petits Ruminants hotspots **(A)**, clusters and outliers **(B)**, and risk map **(C)** based on overall cumulative incidence.

There was a strong global clustering of PPR cases indicated by a significant Moran's I: 0.49 (*Z*-score = 7.66, *P* < 0.01) in 2014, 0.16 (*Z*-score = 1.98, *P* = 0.04) in 2015, 0.22 (*Z*-score = 2.55, *P* = 0.01) in 2016, and 0.26 (*Z*-score = 2.93, *P* < 0.01) in 2017. Figures 1–5 show PPR hotspots, high–high, low–low clusters, and predicted cumulative incidence per 10,000 population in 2014, 2015, 2016, and 2017 and for all 4 years together, respectively. Nine districts (Nawabganj, Naogaon, Rajshahi, Nator, Bogra, Noakhali, Faridpur, Madaripur, and Moulvibazar) had high–high (high numbers of cases in a district with high numbers of cases in surrounding districts) local PPR clusters (Figures 1B, 2B, 3B, 4B, 5B). Naogaon and Mulvibazar districts showed high–high clusters frequently (Figures 2B, 3B, 4B, 5B). We identified low–low (low numbers of cases in a district with low numbers of cases in surrounding districts) PPR clusters in 17 districts mostly in four divisions in Bangladesh: Jhalokathi, Barguna, Barisal, Patuakhali (Barisal division), Bagerhat, Khulna, Kusthia, Magura, Chuadanga (Khulna division), Manikganj, Faridpur, Gopalganj, Madaripur (Dhaka division), Feni, Lakshipur, Cox's Bazar (Chittagong division), and Mymensingh (Figures 1B, 2B, 3B, 4B, 5B). Only one low–high (low numbers of cases in a district with high numbers of cases in surrounding districts) outlier was detected in 2017. Peste des Petits Ruminants hotspots were detected in 9 districts: Joypurhat, Sylhet, Chittagong, Nator, Feni, Rajshai, Naogaon, Bogra, and Bandarban. Joypurhat, Sylhet, and Chittagong districts exhibited hotspots frequently (Figures 1A, 2A, 3A, 4A, 5A).

**Figure 6:**
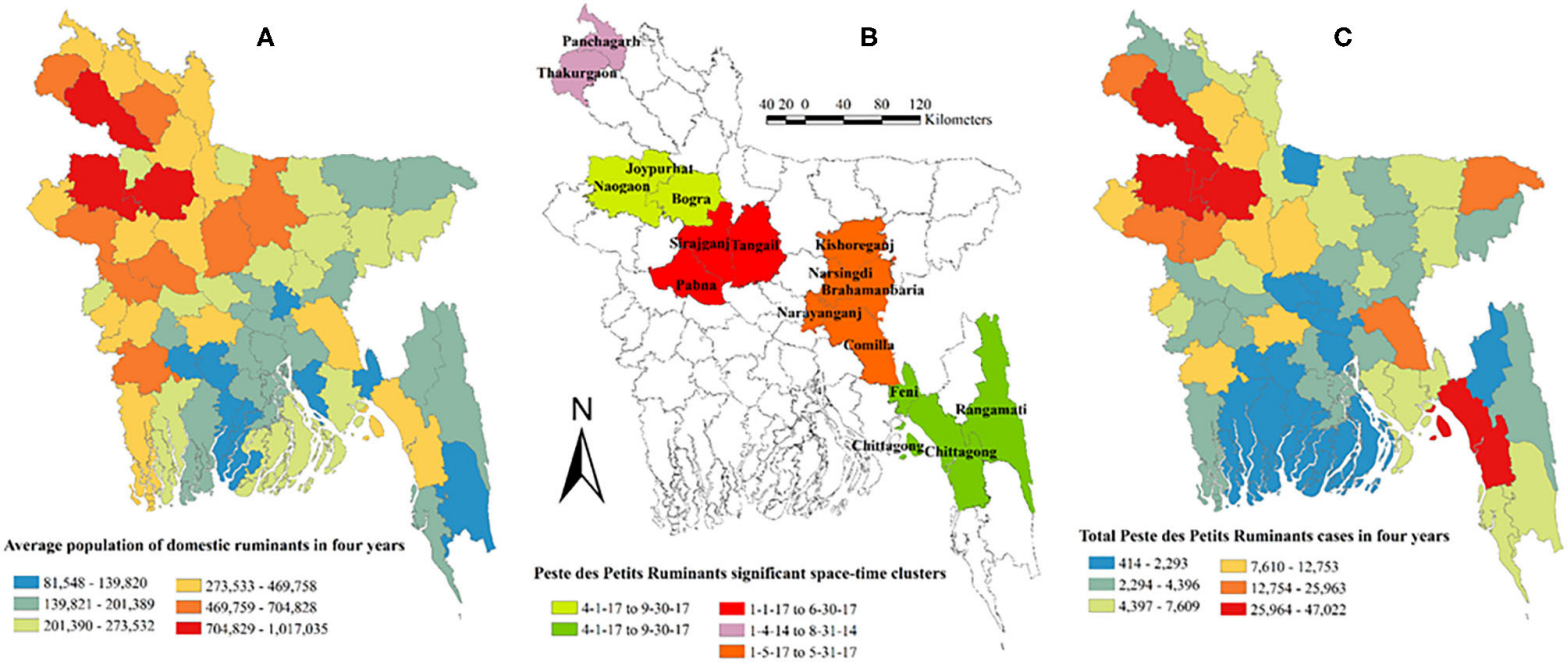
Map of Bangladesh showing average population of domestic ruminants in 4 years, PPR significant space-time clusters, and total PPR cases in 4 years. **(A)** Average population of domestic ruminants in 4 years. **(B)** Peste des Petits Ruminants significant space-time clusters. **(C)** Total Peste des Petits Ruminants cases in 4 years.

We identified five space-time clusters that included 17 districts in five divisions. Four out of five clusters were detected in 2017. Most clusters occurred between January and August (Table 2). Figure 6 shows maps of the small ruminant population, significant space-time clusters for PPR, and total number of PPR cases in small ruminants in Bangladesh.

**Table 2:** Significant space-time clusters of Peste des Petits Ruminants cases reported from 64 districts in Bangladesh between January 2014 and December 2017.

**Districts**	**Radius (km)**	**O/E**	**LLR**	**Time period**	***P-*value**
Joypurhat, Naogaon, Bogra	42.1	4.5	21,650.5	April 1, 2017–September 30, 2017	<0.001
Chittagong, Rangamati, Feni	75.8	6.1	12,700.5	April 1, 2017–September 30, 2017	<0.001
Sirajganj, Tangail, Pabna, Natore	51.9	2.6	5,717.0	January 1, 2017–June 30, 2017	<0.001
Panchagarh, Thakugaon	40.2	2.5	1,909.0	January 1, 2014–August 31, 2014	<0.001
Brahamanbaria, Narsingdi, Kishoreganj, Narayanganj, Comilla	57.6	3.5	1,180.9	May 1, 2017–May 31, 2017	<0.001

*Maximum spatial window = 10% of study area. Maximum temporal window = 6 months*.

*LLR, Log likelihood ratio; O/E, observed/expected*.

The maps of predicted PPR cumulative incidence, the hotspots and clusters in each of the respective years were similar. Higher cumulative incidences of PPR were predicted in north-west, north-east, and south-east parts of Bangladesh in each year and all 4 years together. In contrast, the lower cumulative incidences of PPR were predicted mostly in south and south-west areas of Bangladesh in each year and all 4 years together (Figures 1C, 2C, 3C, 4C, 5C).

### Spatial Risk Factors

**Figure 7:**
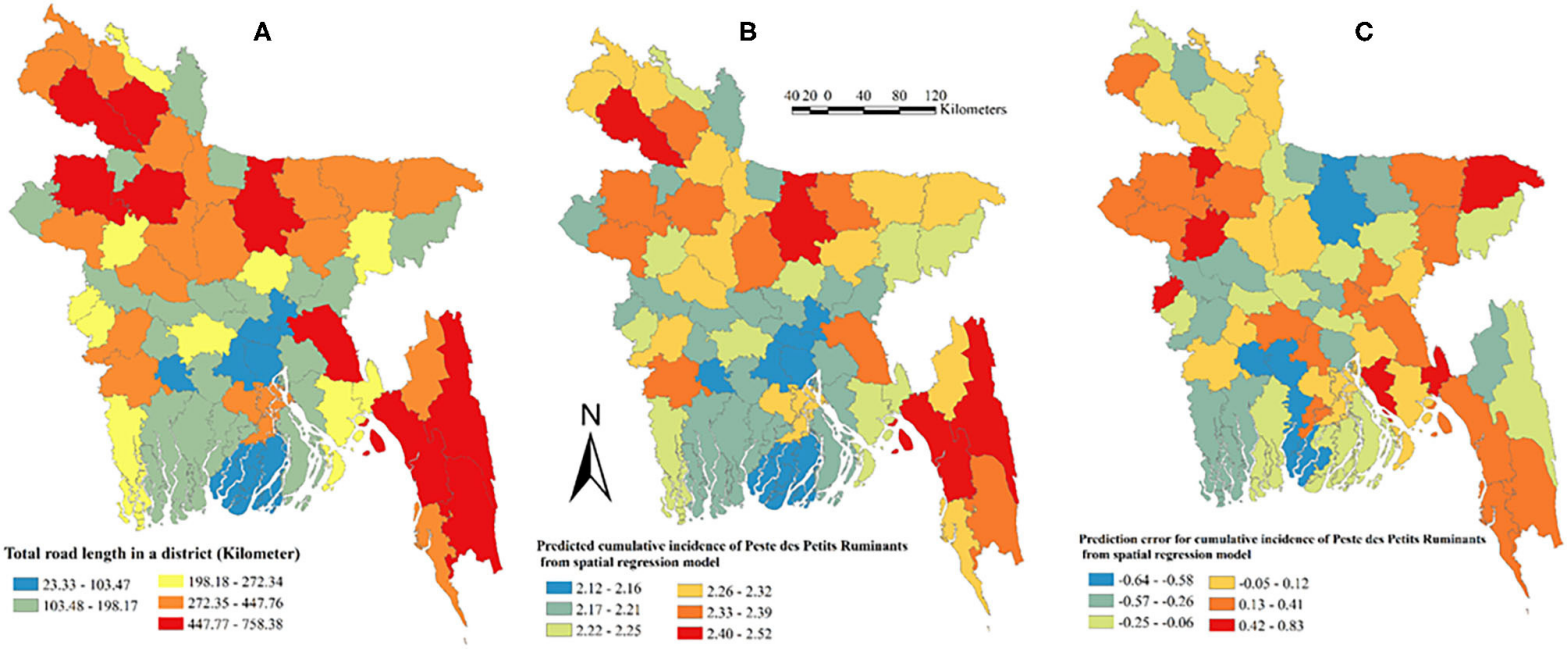
Map of Bangladesh showing total road length, prediction error, and predicted cumulative incidence of PPR from the geographically weighted regression model. **(A)** Total road length in a district. **(B)** Predicted cumulative incidence of Peste des Petits Ruminants from geographically weighted regression model. **(C)** Prediction error.

The non-significant Jarque-Bera test result indicated normality of the error in ordinary least squares (OLS) regression analysis. A non-significant Breusch-Pagan test result indicated constant variance of the dependent variable around the regression line. A highly significant (*P* < 0.001) Moran's *I*-test result indicated spatial dependence. A spatial-lag model was selected for univariable and multivariable GWR analyses based on these results of OLS regression. In univariable GWR analysis, road length and waterbodies were associated (*P* ≤ 0.10) with PPR incidence in Bangladesh (Table 3). In the final multivariable GWR model, road length (estimate = 0.0005; SE = 0.0003, *P* = 0.03) was positively associated with PPR incidence in small ruminants in Bangladesh. This indicates that for every 1 km increase in the road length, the PPR cumulative incidence increased by 5 per 10,000 small ruminants. The final model had an *R*-squared value of 0.27. Total road length (km) in every district, predicted error, and predicted PPR cumulative incidence in small ruminants in Bangladesh are shown in Figure 7. The road length per district varied from 22 to 758 km. The maximum road length (448–758 km) was present in Rangpur, Dinajpur, Naogaon, Bogra, Mymensingh, Comilla, Chittagong, Rangamati, and Bandarban districts. The highest predicted PPR cumulative incidence per district were observed in Chittagong, Rangamti, Mymensingh, and Dinajpur districts.

**Table 3:** Explanatory variables associated with log-transformed overall cumulative incidence of Peste des Petits Ruminants in Bangladesh using univariable geographically weighted regression analysis.

**Variables**	**Categories**	**Coefficient**	**SE**	***P*-value**
Solar radiation (kJ m^−2^)	Winter	−0.00007	0.0001	0.6207
	Pre-monsoon	0.0002	0.0001	0.1252
	Monsoon	0.0002	0.0001	0.3464
	Post-monsoon	0.0001	0.0003	0.7110
Temperature (°C)	Winter	−0.0484	0.1023	0.6357
	Pre-monsoon	−0.1027	0.0888	0.2476
	Monsoon	−0.0274	0.1254	0.8273
	Post-monsoon	−0.1309	0.1377	0.3420
Precipitation (mm)	Winter	−0.0230	0.0216	0.2864
	Pre-monsoon	0.0011	0.0015	0.4332
	Monsoon	0.0007	0.0005	0.1557
	Post-monsoon	0.0019	0.002	0.4347
Wind speed (m s^−1^)	Winter	0.0341	0.2841	0.9045
	Pre-monsoon	−0.0811	0.1805	0.6531
	Monsoon	−0.0878	0.1451	0.5448
	Post-monsoon	−0.0407	0.1729	0.8662
Average population of cattle and buffaloes	–	0.00005	0.000003	0.1451
Elevation (m)	–	−0.0002	0.0016	0.8913
Length of river (km)	–	0.0003	0.0002	0.1133
Area of inland water bodies (km^−2^)	–	−0.00005	0.00008	0.5511
Length of road (km)	–	0.0005	0.0003	0.0393
Length of rail road (km)	–	0.0005	0.0005	0.3866
Land cover	Tree-cover, broadleaved, evergreen	0.0002	0.0014	0.8915
	Tree-cover, broadleaved, deciduous, closed	0.0027	0.0025	0.2706
	Tree-cover, regularly flooded, saline water	0.000007	0.0002	0.9624
	Mosaic: tree-cover and other natural vegetation	−0.0006	0.0010	0.5654
	Tree-cover, burnt	0.00003	0.00007	0.6162
	Shrub-cover, closed-open, evergreen	0.00008	0.0003	0.8357
	Shrub-cover, closed-open, deciduous	−0.0665	0.1540	0.6659
	Cultivated and managed areas	0.00003	0.00003	0.3694
	Mosaic: cropland, tree-cover, other natural vegetation	−0.00001	0.0002	0.9401
	Artificial surface and associated areas	0.00009	0.0008	0.9241
	Water bodies	−0.0004	0.0002	0.0654

## Discussion

We have described for the first time the hotspots, clusters in space and time, and environmental risk factors for PPR in small ruminants in Bangladesh based on 4 years of national-level passive surveillance data. Some high–high clusters and hotspots were identified in Rajshahi, Chittagong, and Sylhet divisions, and the distribution of PPR in small ruminants in Bangladesh was found to be associated with road length in a district. The results suggest that a risk-based vaccination strategy can be considered the first step toward PPR eradication from Bangladesh.

About 5.2 million PPR cases were reported in small ruminants in Bangladesh during the 4-year study period. The actual number of PPR cases in small ruminants would be much higher than that reported here because not all farmers have access to upazila veterinary hospitals, and the criterion used to define cases and maximize positive predictive value. The highest number of cases were reported in the monsoon season and in Rajshahi division. The higher occurrence of PPR in the monsoon season has also been reported by other authors (15, 27, 28). The rainy season is a stressor for small ruminants, which might explain the higher occurrence of PPR at this time of the year. Moreover, during the monsoon season, small ruminants are mostly kept indoors and therefore they remain in close contact, which further facilitates the transmission of highly contagious PPR virus. Four districts (Joypurhat, Naogaon, Nawabganj, and Rajshahi) in Rajshahi division share a common border with India. These districts also showed high–high clusters and hotspots in different years. Cross-border animal movement occurs from India to Bangladesh but mostly involves cattle and buffalo. A small number of sheep also are imported, but there are no reports of goats imported from India (29). Subclinically infected buffalo and sheep coming from India might increase the risk of PPR transmission in these districts. However, molecular evidence of the PPRV isolates in Bangladesh and India also suggest transboundary transmission (30–33).

Peste des Petits Ruminants is a vaccine-preventable disease, and safe, highly efficacious attenuated live vaccine is available. Usually, a lifelong immune response is induced after vaccination and after recovery from PPR, but to ensure a protective immunity level is maintained, revaccination is recommended every 3 years (6, 34). In Bangladesh, vaccine production is insufficient (~5.0 million doses). Implementation of a mass vaccination program has been found to be protective in some goat-rearing districts where animal movement is predominant (35). There is a global goal of PPR eradication by 2030, based on four steps: 1—assessment, 2—control, 3—eradication, and 4—post-eradication follow-up (8). Vaccination of all animals older than 3 months of age is suggested in stage 2 (8). The risk of PPR has been reported to be significantly higher in winter and monsoon seasons in older animals (4–24 months) and in Jamunapari goats (15). We identified 14 hotspots and high–high cluster districts: Joypurhat, Nawabganj, Naogaon, Rajshahi, Nator, Bogra (Rajshahi division), Faridpur, Madaripur (Dhaka division), Moulvibazar, Sylhet (Sylhet division), Noakhali, Chittagong, Feni and Bandarban (Chittagong division). In our resource-limited setting, the first step in control might be vaccinating high-risk animals in these hotspots and high–high clusters before winter and monsoon seasons (16, 36).

Seventeen low–low PPR clusters were detected in our study: Jhalokathi, Barguna, Barisal, Patuakhali (Barisal division), Bagerhat, Khulna, Kusthia, Magura, Chuadanga (Khulna division), Manikganj, Faridpur, Gopalganj, Madaripur (Dhaka division), Feni, Lakshipur, Cox's Bazar (Chittagong division), and Mymensingh. Due to limited resources, we do not recommend to initiate vaccination in these districts—except for Faridpur, Madaripur, and Feni (because they were also identified as hotspots and high–high clusters in 1 year). However, control of animal movements from high risk areas to these low risk areas, proper nutrition, best practice biosecurity management, and introducing healthy animals to herds will help reduce the risk further.

Road length was found to be significantly associated with PPR cumulative incidence in Bangladesh. Animal movement is the most important factor in the spread of transboundary animal diseases as reflected by the road length as a risk factor in our study. Other authors (37–39) have also reported similar findings. The movement of animals normally occurs *via* supply chain networks (herds-local small and big market-central market). Goat meat is very popular among Bangladeshi people. For that, around 15 million goats are slaughtered annually in Bangladesh, of which 40% are slaughtered during one religious festival known as “Eid-ul-Adha” (40). Huge numbers of small and large livestock markets are held before “Eid-ul-Azha” which also facilitates the transmission of PPRV along the supply chain. Animals are transported mainly *via* the road and river networks. Transportation vehicles contaminated by PPRV from the excretions of infected animals can also transmit the infection from one place to another. During festivals and year-round, movement of animals is difficult to control. However, control of PPR using strategic vaccination as described above together with increased awareness through participatory training and education of livestock producers and restriction of animal movements from hotspots and high–high clusters to low–low cluster areas might be the first step toward PPR eradication from Bangladesh. As Rangpur, Dinajpur, Naogaon, Bogra, Mymensingh, Comilla, Chittagong, Rangamati, Bandarban, Rajshahi, Noakhali, Moulvibazar, Natore, Feni, Faridpur, and Sylhet have more road length, PPR control program should be prioritized in these districts.

Upazila (subdistrict) veterinary hospitals are located throughout Bangladesh, and an upazila has several (average 10) unions. Farmers living close to veterinary hospitals or that have access to a good road network physically take their animal to a hospital location for the diagnosis to be made. However, other farmers in remote areas rely on community-based livestock service providers. So, it is plausible that in an upazila with more roads cases might be more likely to be reported. We recommend using additional surveillance resources to target upazilas that have transportation challenges—e.g., provision of ambulatory services and other forms of active surveillance.

Although we studied a large volume of 4 years PPR data, our study has some limitations. Peste des Petits Ruminants is mostly diagnosed by clinical signs and infrequently by laboratory confirmation. However, we included only those cases which have all of the characteristic clinical signs of PPR to increase the positive predictive value of presumptive diagnosis. We also note that some laboratory conformation of PPR cases occurs in Bangladesh. Moreover, active surveillance for PPR does not exist, and there is limited control using vaccination in Bangladesh. In this scenario, passive surveillance data can also generate valuable knowledge about hotspots and clusters, which are essential to disease control decisions. The distribution of PPR cases according to species, breed, age, and gender were not available, although this information is usually collected for each case before being aggregated. Within country and transboundary animal movement data, data on vaccination coverage in each district and live animal market data were not available; these are all important factors in the epidemiology of PPR. Thorough analysis of passive surveillance data at the national level can create important knowledge about diseases to enable disease control decisions. The DLS does not have a sufficiently large enough work force to collect animal health and disease information at the farm level. However, improvement of disease data collection at the upazila (subdistrict) level would be a valuable asset in terms of exploring the epidemiology of endemic diseases such as PPR.

We identified spatiotemporal clusters (hotspots, high–high, low–low clusters) and the influence of environmental risk factors on the epidemiology of PPR in Bangladesh. Spatiotemporal clusters should be selected for vaccination in future eradication programs in Bangladesh. We recommend that additional information should be included in the DLS passive surveillance network (e.g., farm address/geographic coordinates, herd size and composition, number of animals vaccinated) and if individual case record ancillary information (e.g., species, age, sex, breed and other animal characteristics), preferably in a digital form.

## Data Availability Statement

The original contributions generated in the study are included in the article/Supplementary Materials, further inquiries can be directed to the corresponding authors.

## Author Contributions

AR, MW, and BM-L: conceptualization and formal analysis. AR and BM-L: software. SI, MS, and MT: investigation. AR, SI, MS, and MT: data curation. AR and SI: writing—original draft preparation. SI, MS, MT, MW, and BM-L: writing—review and editing. MW and BM-L: supervision. BM-L: funding acquisition. All authors: have read and agreed to the published version of the manuscript.

## Conflict of Interest

The authors declare that the research was conducted in the absence of any commercial or financial relationships that could be construed as a potential conflict of interest.
